# Decline in the Incidence of Chronic Subdural Hematoma During the Coronavirus Disease 2019 Pandemic: A Retrospective Single-Center Descriptive Study

**DOI:** 10.3389/fneur.2022.865969

**Published:** 2022-05-13

**Authors:** Ryosuke Maeoka, Ichiro Nakagawa, Keigo Saeki, Hiroyuki Nakase, Hideyuki Ohnishi

**Affiliations:** ^1^Department of Neurosurgery, Ohnishi Neurological Center, Hyogo, Japan; ^2^Department of Neurosurgery, Nara Medical University, Nara, Japan; ^3^Department of Epidemiology, Nara Medical University School of Medicine, Nara, Japan

**Keywords:** head trauma, chronic subdural hematoma, coronavirus disease 2019, pandemic, stay-at-home

## Abstract

The coronavirus disease 2019 (COVID-19) pandemic has forced restrictions on social activities in some areas. There has also been a decrease in the number of trauma patients in the United States during the COVID-19 pandemic. Chronic subdural hematoma (CSDH) is a traumatic disorder that often develops following head injury. We therefore investigated the impact of the COVID-19 pandemic on CSDH. In this retrospective single-center descriptive study from April 2018 through September 2021, there were 5,282 head trauma patients and 196 patients with CSDH in the pre-pandemic group compared to 4,459 head trauma patients and 140 patients with CSDH in the intra-pandemic group. Significant decreases in the incidence rate (IR) of head trauma (951/100,000 vs. 795/100,000 person-years; IR ratio (IRR): 0.836, 95% confidence interval (CI): 0.803–0.870, *p* < 0.001) and also in the IR of CSDH (35.0/100,000 vs. 24.8/100,000 person-years, IRR: 0.708, 95% CI: 0.570–0.879, *p* = 0.002) were seen in the intra-pandemic group compared to the pre-pandemic group. In this study, the COVID-19 pandemic was associated with significant decreases in the IRs of head trauma and CSDH due to forced restrictions on social activities. Besides, the IR of mild cases of CSDH was significantly lower in the intra-pandemic group than in the pre-pandemic group (IRR: 0.68, 95% CI: 0.51–0.89, *p* = 0.006). Fewer people being out in communities should result in fewer chances for head trauma and CSDH. On the other hand, forced restrictions on social activities due to the COVID-19 pandemic should aggravate CSDH.

## Introduction

Chronic subdural hematoma (CSDH) is a common neurosurgical disorder that mainly affects elderly individuals (1). With the demographic shift toward an aging population, incidence rates (IRs) of CSDH have been rising (2). The IR of CSDH among United States veterans and elderly individuals between 2000 and 2012 was reported as 79.4/100,000 person-years (3). Recently, the initial trauma has been considered as the first step in the pathogenesis of CSDH (2, 4). CSDH is therefore considered a traumatic disorder that often develops after head injury. In Japan, the first patient with coronavirus disease 2019 (COVID-19) was reported on January 16, 2020. To control the viral spread, numerous government agencies across several areas of many prefectures enforced restrictions with stay-at-home orders, impacting social activities, including travel, going out, eating out, and drinking outside. Government agencies in Japan implemented stay-at-home orders for some prefectures, including Akashi city, Hyogo, from April 7, 2020. The COVID-19 pandemic has resulted in a restructuring of the healthcare systems worldwide and has forced restrictions on social activities, and declines in trauma have been reported at local, regional, and national levels (5–8). In fact, significant declines in physical activity during the COVID-19 pandemic have been reported among the elderly in Japan (9, 10). Moreover, some researchers have reported that regular physical activity is associated with an increased incidence of activity-related injury (11–13). We therefore hypothesized that social restrictions would be associated with a significantly lower IR of head trauma and would have resulted in a decline in the IR of CSDH. On the other hand, CSDH patients were reported to present with a longer interval from the initial head injury to the final diagnosis in the lockdown period than in the same period the previous year (14). We also hypothesized that social restrictions would change the severity of CSDH.

## Methods

This retrospective single-center descriptive study in the Akashi city, Hyogo, Japan involved a total of 303,899 inhabitants in the Akashi region as population in the pre-pandemic period. This study also involved a total of 304,553 in the Akashi region as population in the intra-pandemic period. In this study, all patients who presented with head trauma and all CSDH treated with burr hole evacuation in our institution in Akashi city, Hyogo, Japan, from April 1, 2018 through September 30, 2021, were reviewed. The electronic medical records of each patient were reviewed, and data were obtained regarding patient's age, sex, use of anticoagulants and antiplatelet medicines, date of surgery, and preoperative modified Rankin Scale (mRS) score to evaluate whether the severity of CSDH had exacerbated because of patients refraining from seeing doctors (15). We defined preoperative mRS score of 1–3 as mild cases and score of 4–5 as severe cases. During the study period, three institutions in Akashi had a department of neurosurgery. According to the information provided by the Akashi City Fire Department, about 70% of patients with head traumas were transported to our institution every month. We conducted a retrospective review of 9,741 consecutive patients with head trauma and 390 patients with CSDH. Inclusion criteria for head trauma were a diagnosis of head trauma with or without head imaging. Inclusion criteria for CSDH were: (1) new diagnosis of CSDH; and (2) radiological findings of CSDH from computed tomography (CT) of the head. Exclusion criteria included: (1) patients with CSDH from other secondary causes, such as post-craniotomy or intracranial hypotension; (2) patients with recurrent CSDH; and (3) patients with initial imaging showing acute subdural hematoma. Preoperative severity was measured by the mRS score. We compared the IRs of CSDH and head trauma and preoperative mRS score between the 21 months prior to the pandemic (April 1, 2018 to December 31, 2019; pre-pandemic group) and the first 21 months of the pandemic (January 1, 2020 to September 30, 2021; intra-pandemic group). Each patient was examined by fellowship-trained neurosurgeons. Written informed consent was obtained from each patient, their nearest relative, or a person who had been given authority to provide consent for admission and surgery for the patient. The institutional ethics committee of the institution approved this study (approval no. 211101).

Categorical variables are provided as numbers (percentage) and continuous variables are reported as median with interquartile range (IQR). Statistical analysis was performed with the chi-squared test for categorical variables and Wilcoxon's rank-sum test for continuous variables. Trends in the IR of CSDH and head trauma were reported as IR ratios (IRRs) with 95% confidence intervals (CIs). Log-linear Poisson regression models have been used to estimate age-adjusted IRRs in each age strata, as crude estimates may give biased results. Two-sided *p*-values of less than 0.05 were considered to indicate statistical significance. Descriptive and frequency analyses were performed, and comparisons were made using JMP version 14.0.0 software (SAS Institute, Cary, NC, USA).

## Results

A total of 9,741 patients presented with head trauma at our hospital during the study period; 5,282 patients (median age, 54 years; IQR: 10–76 years; 2,719 [51.5%] men) were in the pre-pandemic group and 4,459 patients (median age, 57 years; IQR: 10–78 years; 2,304 [51.7%] men) were in the intra-pandemic group. Significant declines in numbers (5,282 vs. 4,459 cases) and IR (951/100,000 vs. 795/100,000 person-years) were seen in the intra-pandemic group compared to the pre-pandemic group. The IRR of head trauma was 0.836 (95% CI: 0.803–0.870; *p* < 0.001) (Table 1). Although no significant difference was seen in sex (*p* = 0.85), the median age of head trauma patients was significantly higher in the intra-pandemic group than in the pre-pandemic group (*p* = 0.02). The IRR of head trauma patients was significantly higher among patients over the age of 70 years than among patients under the age of 20 years or 20–70 years (*p* < 0.001) (Table 1).

**Table 1:** Incidence of head trauma according to pre- and intra-pandemic status in Akashi city, Japan.

	**Pre-pandemic status Apr 1, 2018 – Dec 31, 2019**			**Intra-pandemic status Jan 1,2020 – Sep 30, 2021**				
	**Cases**	**Person-years**	**IR^**a, †**^**	**Cases**	**Person-years**	**IR^**a, †**^**	**IRR^**b, §**^(95%CI^**c**^)**	***P* value**
All	5,282	555,488	950.9	4,459	561,207	794.5	0.84 (0.80–0.87)	<0.001
0–19 years	1,780	100,606	1,769.3	1,464	101,348	1,444.5	0.82 (0.76–0.87)	<0.001
20–69 years	1,551	356,773	434.7	1,219	353,876	344.5	0.79 (0.74–0.85)	<0.001
70+ years	1,951	98,110	1,988.6	1,776	105,983	1,675.7	0.84 (0.79–0.90)	<0.001

a *IR, incidence rate*;

b *IRR, incidence rate ratio*;

c *CI, confidence interval*.

† *per 100,000 person-year*.

§ *IR in intra-pandemic status/ IR in pre-pandemic status*.

A total of 390 patients with CSDH underwent burr hole evacuation during the study period. Fifty-four patients were excluded, comprising 43 patients with recurrent CSDH, 9 patients with iatrogenic CSDH, and 2 patients with CSDH related to cerebrospinal fluid hypovolemia. A final total of 336 patients were included in this study. Following the exclusion criteria, 196 patients (median age, 80 years; IQR: 72.5–85 years; 135 [68.9%] men) in the pre-pandemic group and 140 patients (median age, 80 years; IQR: 74–85 years; 90 [64.3%] men) in the intra-pandemic group were included for analysis. Significant declines in numbers (196 vs. 140 cases) and IR of CSDH (35.0/100,000 vs. 24.8/100,000 person-years) were seen in the intra-pandemic group compared to the pre-pandemic group. The IRR of CSDH was 0.708 (95% CI: 0.570–0.879, *p* = 0.002) (Table 2). However, we saw no significant differences in age (*p* = 0.66), sex (*p* = 0.38), and rates of taking anticoagulants (15 patients [7.7%] vs. 15 patients [10.7%]; *p* = 0.33) and antiplatelet medicines (31 patients [15.8%] vs. 22 patients [15.7%]; *p* = 0.98) between the pre- and intra-pandemic groups. No significant difference was seen in the median preoperative mRS score (3 [IQR: 2–4] vs. 3 [IQR: 2–4]; *p* = 0.87) between the pre- and intra-pandemic groups. The IR for each mRS strata of patients with mRS score of 3 was significantly lower in the intra-pandemic group than in the pre-pandemic group (IRR: 0.58, 95% CI: 0.38–0.87, *p* = 0.008). Declines in IR were seen in both mild and severe cases of CSDH. Although the IR in the mild cases of CSDH was significantly lower in the intra-pandemic group than in the pre-pandemic group (IRR: 0.68, 95% CI: 0.51–0.89, *p* = 0.006), for the severe cases of CSDH, the 95% CI for the IRR included 1 (Table 2).

**Table 2:** Incidence of chronic subdural hematoma according to pre- and intra-pandemic status in Akashi city, Japan.

	**Pre-pandemic status Apr 1, 2018 – Dec 31, 2019**			**Intra-pandemic status Jan 1, 2020 – Sep 30, 2021**				
**Characteristics**	**Cases**	**Person-years**	**IR^**a, †**^**	**Cases**	**Person-years**	**IR^**a, †**^**	**IRR^**b, §**^(95%CI^**c**^)**	***P* value**
All	196	559,914	35.0	140	564,969	24.8	0.71 (0.57–0.88)	0.002
0–19 years	1	102,161	1.0	3	102,624	2.9	2.99 (0.31–28.7)	0.34
20–69 years	31	358,076	8.7	18	354,912	5.1	0.59 (0.33–1.05)	0.071
70+ years	164	99,525	164.8	119	107,210	111.0	0.67 (0.53–0.85)	0.001
Preoperative mRS ^d^								
1	18	559,914	3.2	14	564,969	2.5	0.77 (0.38–1.55)	0.47
2	43	559,914	7.7	34	564,969	6.0	0.78 (0.50–1.23)	0.29
3	62	559,914	11.1	36	564,969	6.4	0.58 (0.38–0.87)	0.008
4	47	559,914	8.4	40	564,969	7.1	0.84 (0.55–1.29)	0.43
5	26	559,914	4.6	16	564,969	2.8	0.61 (0.33–1.14)	0.12
1–3	123	559,914	22.0	84	564,969	14.9	0.68 (0.51–0.89)	0.006
4–5	73	559,914	13.0	56	564,969	9.9	0.76 (0.54–1.08)	0.12

a *IR, incidence rate*;

b *IRR, incidence rate ratio*;

c *CI, confidence interval*;

d *mRS, modified Rankin scale*.

† *per 100,000 person-year*.

§ *IR in intra-pandemic status/ IR in pre-pandemic status*.

## Discussion

This retrospective single-center descriptive study revealed that the COVID-19 pandemic correlated with significant declines in both the number and IR for hospital presentation due to head trauma (−16.4%) and CSDH (−29.2%) during the intra-pandemic period, compared to the pre-pandemic period. Although there are some reports of declines in trauma at local, regional, and national levels during the COVID-19 pandemic period, this represents the first study to examine associations between the COVID-19 pandemic and CSDH, as far as we know (5–8).

The IR of head trauma in all three age strata declined significantly. The IRR of head trauma (adjusted to age under 20 years) for patients aged more than 70 years was highest among the three age strata. Although the forced restrictions on social activities, such as stay-at-home orders and lockdowns, were enacted, resulting in unprecedented limitations on public and commercial interactions, the effects of these restrictions on head trauma appear particularly prominent in the lower age strata of under 70 years. As a result, a significant increase in the median age of head trauma patients was seen during the intra-pandemic period. In addition, a significant decline in physical activity during the COVID-19 pandemic period has been reported among the elderly in Japan (9, 10). Regular physical activity has been associated with an increased incidence of activity-related injuries (11–13). Although declines in physical activity could result in declining head trauma among the elderly, the effects of restricting social activities on head trauma were relatively small in the population aged over 70 years. We attribute this to head trauma occurring at places unrelated to social activities, such as indoors, particularly in the elderly (16). We consider that this is why the effects of restricted social activities on head trauma were small among the elderly.

CSDH is considered a traumatic disorder that often develops after head injury. Association between CSDH and minor head trauma, such as whiplash trauma and indirect trauma to the cranium, have been reported in elderly individuals with brain atrophy (2, 17). We observed significant declines in the number and IR of CSDHs between pre- and intra-pandemic groups. As more than three-quarters of CSDH cases in both pre- and intra-pandemic groups occurred in patients over the age of 72 years, no major difference in the age of CSDH patients was identified between groups. Declines in the number of head traumas may result in declines in the number of CSDHs in the elderly. Chan et al. reported that CSDH patients presented with longer interval, from the initial head injury to the final diagnosis, in the lockdown period than in the same period in the previous year (14). On the other hand, they also found no difference in the severity of CSDH between the lockdown period and the same period in the previous year (14). However, this study found that the IR in preoperative mRS score of only the mild cases of CSDH in the intra-pandemic period was significantly lower than that in the pre-pandemic period. Consequently, the IR of the severe cases of CSDH in intra-pandemic relatively increased. We consider that the forced restrictions on social activities due to COVID-19 pandemic result in CSDH patients refraining from seeing doctors and hence aggravates CSDH.

The present study has some limitations. First, we did not include outpatients with CSDH, who had not undergone burr hole evacuation. Second, this study focused on a single center. There is a certainty that patients who were treated in other health systems and trauma centers were excluded. This study also included a small number of patients. The results of this analysis may not reflect a global decline in the IR of CSDH. Finally, trauma by its nature often shows wide variations from month to month, and so other factors may have affected declines in the numbers of head traumas and CSDHs.

We identified significant associations between the COVID-19 pandemic and declines in the numbers and IRs of head traumas and CSDHs. Fewer people being out in their communities may result in fewer chances for head trauma and CSDH. According to this study, forced restrictions on social activities due to COVID-19 pandemic results in CSDH patients refraining from seeing doctors, and aggravates CSDH. We would like to raise alarm for this situation, and clinicians must consider this insidious and curable neurosurgical disorder.

## Data Availability Statement

The raw data supporting the conclusions of this article will be made available by the authors, without undue reservation.

## Ethics Statement

The studies involving human participants were reviewed and approved by the Institutional Ethics Committee of the Ohnishi Neurological Center (Approval No. 211101). Written informed consent to participate in this study was provided by the participants' legal guardian/next of kin.

## Author Contributions

RM and IN contributed to the study design and conception, drafting the manuscript, and editing of the manuscript. RM and HO collected the clinical data. RM, IN, and KS contributed to clinical data analysis and interpretation. KS conducted the statistical analysis. HN and HO supervised all aspects of this study. RM took responsibility for the study as a whole. All authors contributed to the article and approved the submitted version.

## Conflict of Interest

The authors declare that the research was conducted in the absence of any commercial or financial relationships that could be construed as a potential conflict of interest.

## Publisher's Note

All claims expressed in this article are solely those of the authors and do not necessarily represent those of their affiliated organizations, or those of the publisher, the editors and the reviewers. Any product that may be evaluated in this article, or claim that may be made by its manufacturer, is not guaranteed or endorsed by the publisher.
